# Measurement of Small-Slope Free-Form Optical Surfaces with the Modified Phase Retrieval

**DOI:** 10.3390/mi13010082

**Published:** 2022-01-04

**Authors:** Xinxue Ma, Jianli Wang, Bin Wang, Xinyue Liu

**Affiliations:** Changchun Institute of Optics, Fine Mechanics and Physics, Chinese Academy of Sciences, Changchun 130033, China; wangjianli@ciomp.ac.cn (J.W.); eatingbeen@hotmail.com (B.W.); liuxinyue@ciomp.ac.cn (X.L.)

**Keywords:** free-form, metrology, surfaces measurement, phase retrieval

## Abstract

In this paper, we demonstrate the use of the modified phase retrieval as a method for application in the measurement of small-slope free-form optical surfaces. This technique is a solution for the measurement of small-slope free-form optical surfaces, based on the modified phase retrieval algorithm, whose essence is that only two defocused images are needed to estimate the wave front with an accuracy similar to that of the traditional phase retrieval but with less image capturing and computation time. An experimental arrangement used to measure the small-slope free-form optical surfaces using the modified phase retrieval is described. The results of these experiments demonstrate that the modified phase retrieval method can achieve measurements comparable to those of the standard interferometer.

## 1. Introduction

With the rapid development of national defense, aerospace, and other fields, the demand for high-precision and high-quality photoelectric products is increasing, and these photoelectric products are gradually developing toward miniaturization. Using free-form surfaces, the imaging quality of the optical imaging system can be greatly improved; the illumination uniformity of the optical illumination system can be evidently improved; and the transmission efficiency of the information transmission system can also be remarkably improved. With the recent advances in optical design and fabrication, the free-form optical surface is commonly used because of its better performance and compactness [1,2,3].

Because the free-form optical surface has more degrees of freedom for correcting optical aberrations, the high precision free-form optical surface metrology remains difficult and is still a challenge [4,5,6]. Therefore, in recent years, many scholars have studied the optical testing methods of free-form surfaces. Thus, a number of metrology methods have been developed [7,8,9], and these methods are roughly divided into contact metrology and non-contact metrology. Because it is easy to scratch the surfaces with contact metrology, non-contact metrology is preferred for high-precision optical surfaces. The recognized interferometer cannot measure the free-form surfaces. This is not only because the standard interferometry has some typical disadvantages, such as high sensitivity to vibrations or temperature fluctuations, hindering its usage to strictly controlled laboratory conditions, but also because the fringes are too dense, and the interference fringes cannot be generated.

In order to solve the problem of free-form optical surface metrology, we introduce a feasible non-contact measurement method called Phase Retrieval (PR), a high-precision method and an alternative to interferometry for optical testing, with the advantages of compactness, low-cost, and a stable system. PR has emerged as a potential solution for free-form surfaces metrology [10,11,12,13]. As PR is a method of wave-front sensing and a simple experimental arrangement in optical metrology, it has been used in system measurement and alignment; it has, for example, been used in the Hubble Space Telescope and the James Webb Space Telescope [14,15]. Besides, PR has also been applied to test spherical mirror surfaces and rotationally symmetric aspherical surfaces [16,17]. As we know, algorithm is the soul of PR. Some PR algorithms have robustness, multiple solutions, and stagnation; for example, the convergence speed of the fastest gradient search in the PR algorithm is not the fastest, and it usually falls into a local minimum. In addition, the conjugate gradient search method in the PR algorithm is more robust than the fastest gradient search method. Thus, we introduce a new PR algorithm, which potentially has the advantage of improving the efficiency of phase recovery in order to solve the limitations of the traditional PR in its iterative uncertainty and slow convergence speed [18,19].

In this paper, we will first introduce the theory and the application of PR and then the improved PR algorithm in Section 2. In Section 3, the results and analysis of the experiment are presented. The conclusion is finally drawn in Section 4.

## 2. Theory of PR

### 2.1. The Principle of PR

**Figure 1:**
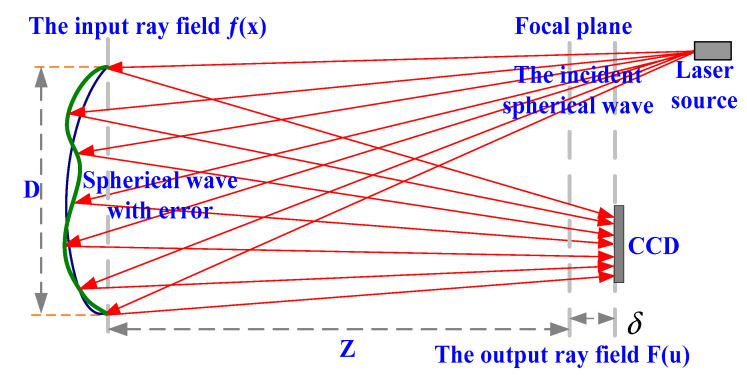
The principle of the Phase Retrieval system.

PR technology is based on the theory that the diffraction of coherent light propagates. PR generally involves estimating a complex-valued phase distribution from known intensity distributions at some properly selected planes. It is an inverse problem in optics, which uses the Fourier transform relationship between the pupil and the in-focus plane to iteratively estimate the phase which is suffering from non-uniqueness. Figure 1 shows the schematic layout of the PR principle. When a beam propagates along the optical axis, the diffraction field distribution is formed at a certain propagation distance. The reference wave emitted from the light source is incident on the measured mirror. After the reflection of the output light field, the complex amplitude distribution of the optical wave front contains the error information of the measured mirror. Using these intensity images and the PR algorithm, one can accurately recover the surface error of the measured mirror [20]. The detector is placed at the focal plane of the wave front reflected or transmitted from the surface under testing and will take a number of images, including those in focus and defocused from the focal plane in both directions. The wave front can be estimated with the known pupil size and the defocus amounts of the detectors [21].

Assuming that the aperture of one measured optical surface is D, the focal length is Z, and the laser wavelength is λ. The generalized pupil function is f(x), whose amplitude is |f(x)|, and the phase is θ, which can be obtained with Zernike polynomial fitting: $\theta (x)=\sum_{n}\alpha_{n}Z_{n}(x)$, where the real number $\alpha_{n}$ represents the first *n*th terms of the polynomial coefficients and $Z_{n}$ indicates the *n*th terms of the Zernike polynomials.

We could get:

(1) f(x)=|f(x)|exp[iθ(x)]

where *x* is an M-dimensional spatial coordinate, and θ is wave-front distortion.

For a linear optical system with the defocus δ in the focal plane, the impulse response function F(u) can be described as:

(2) F(u)=|F(u)|exp[iψ(u)]=ℱ{|f(x)|exp[ε(x,δ)]}

where x is the spatial coordinate of the pupil, u is the coordinates of the image and both of them are two-dimensional vector field coordinates. ψ is the phase of the impulse response, ℱ is the two-dimensional Fourier transform, and ε(x,δ) is the wave-front aberration caused by the defocus δ.

For one PR system, |f(x)| in Equation (1) is the priori condition of a known optical system corresponding to the size and shape of the pupil. ${\left|F(u)\right|}^{2}$ is the image collected by the detector. We estimate $\alpha_{n}$ and then obtain θ in Equation (1) with a number of measurements at different defocuses.

### 2.2. The Modified Gradient Search Algorithm of PR

In this paper, we present a modified gradient search algorithm to solve the phase recovery problem [22,23]. Let $g_{m,k},\theta_{m,k},G_{m,k},\varphi_{m,k}$ be the estimated values of f,δ,F,ψ when the *m*th images iterate *k* times, $g_{k}$ represents the combined estimate value with every $g_{m,k}$ to f when iterated *k* times, which is $g_{k}(x)=\frac{1}{M}\sum_{m=1}^{M}g_{m,k}(x)$.

The initialization is:

- $\theta_{m,k}=0$, $\varepsilon_{m}(x)=\varepsilon (x,\delta_{m})=(\frac{\pi \delta_{m}{\left\|x\right\|}^{2}}{\lambda Z^{2}})$, $g_{k}(x)=\left|f(x)\right|$, m∈[1,M],
- $G_{m,k}(u)=\left|G_{m,k}(u)\right|\exp[i\varphi_{m,k}(u)]=ℱ\{g_{k}(x)\exp[i\varepsilon_{m}(x)]\}$, m∈[1,M],
- $G_{m,k}{}^{\prime }(u)=\left|F(u)\right|\exp[i\varphi_{m,k}(u)]$, m∈[1,M],
- $g_{m,k}{}^{\prime }(x)=\left|g_{m,k}{}^{\prime }(x)\right|\exp[i\theta_{m,k}{}^{\prime }(x)]={ℱ}^{-1}[G_{m,k}{}^{\prime }(u)]\exp[-\varepsilon_{m}(x)]$, m∈[1,M],
- $g_{m,k+1}(x)=\left|f(x)\right|\exp[i\theta_{m,k+1}(x)]=\left|f(x)\right|\exp[i\theta_{m,k}{}^{\prime }(x)]$, m∈[1,M],
- $g_{k+1}(x)=\frac{1}{M}\sum_{m=1}^{M}g_{m,k+1}(x)$.

Repeat from steps b to steps f until the extrusion of the condition, which is the limitation of the iteration times or the function of the object descended to the appointed value. The function of the object is described as [24]:

$$B_{k}=E_{FK}^{2}=N^{-2}\sum_{m=1}^{M}\sum_{u}{\left|G_{m,k}(u)-G_{m,k}{}^{\prime }(u)\right|}^{2}$$

where *N* represents the width of the collected images. According to b and c, the phase $G_{m,k}(u)$ and the phase $G_{m,k}{}^{\prime }(u)$ are equal, so we can get:

(3) $$B_{k}=E_{FK}^{2}=N^{-2}\sum_{m=1}^{M}\sum_{u}{\left[\left|G_{m,k}(u)\right|-\left|F(u)\right|\right]}^{2}$$

We apply the mathematical optimization method with Equation (3) as the function of the object and the unknown quantity about each partial derivative together with the substitution gradient search algorithm, finally obtaining the estimation of the wave-front distortion corresponding to θ, when $B_{k}$ is smallest. The most important application of the gradient search algorithm is the correct description of the function of the object and the partial derivatives of each variable. We first discuss the partial derivative g(x), which is as the unknown variables. We get the derivative from *B* to g(x), respectively, and get the derivative from $B_{k}$ to the real part of $\partial g_{real}$ and the imaginary part of $\partial g_{imag}$

(4) $$\begin{array}{l} \partial g_{real}B_{k}\equiv \frac{\partial B_{k}}{\partial g_{real.k}(x)}=2N^{-2}\sum_{m=1}^{M}\sum_{u}\left[\left|G_{m,k}(u)\right|-\left|F(u)\right|\right]\frac{\partial \left|G_{m,k}(u)\right|}{\partial g_{real,k}(x)}\\ \partial g_{imag}B_{k}\equiv \frac{\partial B_{k}}{\partial g_{imag.k}(x)}=-i2N^{-2}\sum_{m=1}^{M}\sum_{u}\left[\left|G_{m,k}(u)\right|-\left|F(u)\right|\right]\frac{\partial \left|G_{m,k}(u)\right|}{\partial g_{imag,k}(x)} \end{array}$$

where

(5) $$\begin{array}{l} \frac{\partial \left|G_{m,k}(u)\right|}{\partial g_{real,k}(x)}=\frac{\partial }{\partial g_{real,k}(x)}\sum_{y}g_{k}(y)\exp[i\varepsilon_{m}(x)]\exp[\frac{-i2\pi uy}{N}]=\exp[i\varepsilon_{m}(x)]\exp[\frac{-i2\pi ux}{N}]\\ \frac{\partial \left|G_{m,k}(u)\right|}{\partial g_{imag,k}(x)}=\frac{\partial }{\partial g_{imag,k}(x)}\sum_{y}g_{k}(y)\exp[i\varepsilon_{m}(x)]\exp[\frac{-i2\pi uy}{N}]=\exp[i\varepsilon_{m}(x)]\exp[\frac{-i2\pi ux}{N}] \end{array}$$

and

(6) $$\begin{array}{l} \frac{\partial \left|G_{m,k}(u)\right|}{\partial g_{real,k}(x)}=\frac{\partial {\left[{\left|G_{m,k}(u)\right|}^{2}\right]}^{1/2}}{\partial g_{real,k}(x)}=\frac{1}{2\left|G_{m,k}(u)\right|}\frac{\partial {\left|G_{m,k}(u)\right|}^{2}}{\partial g_{real,k}(x)}=\frac{G(u)\exp[-i\varepsilon_{m}(x)+i2\pi ux/N]}{2\left|G(u)\right|}+c.c.\\ \frac{\partial \left|G_{m,k}(u)\right|}{\partial g_{imag,k}(x)}=\frac{\partial {\left[{\left|G_{m,k}(u)\right|}^{2}\right]}^{1/2}}{\partial g_{imag,k}(x)}=\frac{1}{2\left|G_{m,k}(u)\right|}\frac{\partial {\left|G_{m,k}(u)\right|}^{2}}{\partial g_{imag,k}(x)}=\frac{G(u)\exp[-i\varepsilon_{m}(x)+i2\pi ux/N]}{2\left|G(u)\right|}+c.c. \end{array}$$

Thus, Equation (4) can be changed to:

(7) $$\begin{array}{l} \partial g_{real}B_{k}=N^{-2}\sum_{m=1}^{M}\sum_{u}\left[G_{m,k}(u)-\left|F(u)\right|G_{m,k}(u)/\left|G_{m,k}(u)\right|\right]=\frac{-iG(u)\exp[-i\varepsilon_{m}(x)+i2\pi ux/N]}{2\left|G(u)\right|}+c.c.\\ \partial g_{imag}B_{k}=-iN^{-2}\sum_{m=1}^{M}\sum_{u}\left[G_{m,k}(u)-\left|F(u)\right|G_{m,k}(u)/\left|G_{m,k}(u)\right|\right]=\frac{-iG(u)\exp[-i\varepsilon_{m}(x)+i2\pi ux/N]}{2\left|G(u)\right|}+c.c. \end{array}$$

where c.c. represents the former plural conjugate.

Using $G_{m,k}{}^{\prime }(u)=\left|F(u)\right|\exp[i\phi_{m,k}(u)],m\in [1,M]$ to define $G_{m,k}{}^{\prime }(u)$, we could get: $G_{m,k}{}^{\prime }(u)=\frac{\left|F(u)G_{m,k}(u)\right|}{\left|G_{m,k}(u)\right|}$.

Thus, Equation (7) can be expressed as:

(8) $$\begin{array}{l} \partial g_{real}B_{k}=2Real\sum_{m}\left[g_{m,k}(x)-g_{m,k}{}^{\prime }(x)\right]\\ \partial g_{imag}B_{k}=2Imag\sum_{m}\left[g_{m,k}(x)-g_{m,k}{}^{\prime }(x)\right] \end{array}$$

We consider θ(x) as the derivative of the unknown value. From Equation (3) we get the derivative from $B_{k}$ to θ(x):

(9) $$\partial_{\theta }B_{k}=\frac{\partial B_{k}}{\partial \theta_{k}(x)}=2N^{-2}\sum_{m}\sum_{u}\left[\left|G_{m,k}(u)\right|-\left|F(u)\right|\right]\frac{\partial \left|G_{m,k}(u)\right|}{\partial \theta_{k}(x)}$$

Because of

(10) $$\frac{\partial \left|G_{m,k}(u)\right|}{\partial \theta_{k}(x)}=\frac{\partial }{\partial \theta_{k}(x)}\sum_{y}\left|f(y)\right|\exp[i\theta (y)]\exp[i\varepsilon_{m}(x)]\exp[\frac{-i2\pi uy}{N}]=ig_{k}(x)\exp[i\varepsilon_{m}(x)]\exp[\frac{-i2\pi ux}{N}]$$

Then, we could get:

$$\frac{\partial \left|G_{m,k}(u)\right|}{\partial \theta_{k}(x)}=\frac{G_{m,k}(u)(-i)g_{k}^{*}(x)\exp[-i\varepsilon_{m}(x)]\exp[i2\pi ux/N]+c.c.}{2\left|G_{m,k}(u)\right|}$$

Thus, we could get:

(11) $$\begin{array}{ll} \partial_{\theta }B_{k} & =\sum_{m}ig_{m,k}{}^{*}(x)[g_{m,k}{}^{\prime }(x)-g_{m,k}(x)]+c.c.\\ & =-2Imag\sum_{m}\left[g_{m,k}{}^{*}(x)g_{m,k}{}^{\prime }(x)\right]\\ & =-2\left|f(x)\right|\sum_{m}\left|g_{m,k}{}^{\prime }(x)\right|\sin[\theta_{m,k}{}^{\prime }(x)-\theta_{m,k}(x)] \end{array}$$

We consider the Zernike coefficient α(x) as the derivation of the unknown value. From Equation (3), we get the derivative from $B_{k}$ to α(x):

(12) $$\frac{\partial B_{k}}{\partial \alpha_{n,k}}=\sum_{x}\frac{\partial B}{\partial \theta_{k}(x)}\frac{\partial \theta_{k}(x)}{\partial \alpha_{n,k}(x)}$$

Take $\frac{\partial \theta_{k}(x)}{\partial \alpha_{n,k}(x)}=\frac{\partial }{\partial \alpha_{n,k}}[\sum_{n=1}^{m}\alpha_{n,k}Z_{n}(x)]=Z_{n}(x)$ into Equation (12). We get the objective function, which is calculated as:

(13) $$\partial_{\alpha_{n}}B_{k}=-2\sum_{m}\sum_{x}\left|f(x)\right|\left|g_{m,k}{}^{\prime }(x)\right|\sin[\theta_{m,k}{}^{\prime }(x)-\theta_{m,k}(x)]Z_{n}(x)$$

With the objective Equation (3) and its impact on the Zernike coefficient derivative Equation (13), we can use the mathematical optimization algorithm, such as Limited-memory BFGS algorithm, to solve various Zernike wave-front coefficient values [25,26,27].

## 3. Experimental Demonstrations

**Figure 2:**
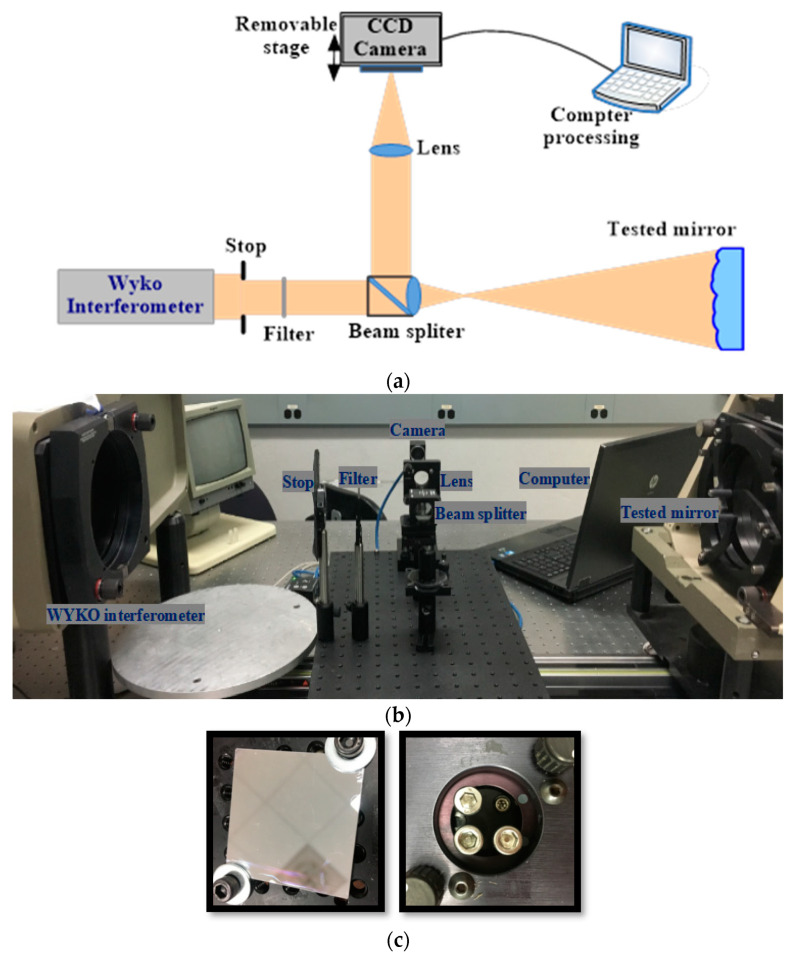
(**a**) The diagram of the measuring installation with the course of the clearance beams; (**b**) the experimental setup of the PR system; and (**c**) the thin measured mirror. The left is the front surface of the thin measured mirror and the right is the back surface of the thin measured mirror. Three screws were used to apply different forces to the measured mirror to change the surface shape.

Here, we demonstrate the measurement ability in small-slope free-form surfaces with the modified phase retrieval discussed in Section 2. Figure 2a shows the diagram of the measuring installation with the course of the clearance beams. In addition, we built the experimental setup to measure the thin, deformable mirror surface (in Figure 2c), shown in Figure 2b. The size of this measured mirror is (35 mm (length) × 35 mm (width) × 15 mm (thickness)), and there are three screws on the back surface of mirror which were used to apply different forces in order to change the surface shape. The collimated laser beam (with the wavelength of 632.5 nm) from the WYKO interferometer passed through the beam splitter and reached the measured surface. The reflected light from the measured surface was directed to the focusing lens, with a focal length of 150 mm, by the beam splitter and then reached the detector. The detector was placed on a computerized moving stage which enabled the detector to take images as it moved away from the focal plane.

**Figure 3:**
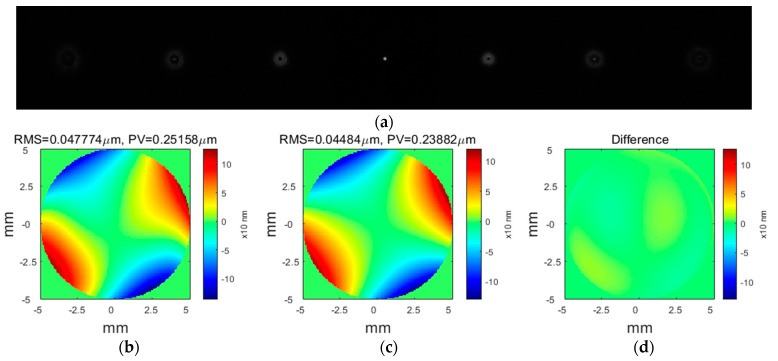
(**a**) Seven 128 pixels * 128 pixels defocused images, from left to right, the defocus is, respectively, −2.2 mm, −1.7 mm, −1.2 mm, 0 mm, 1.2 mm, 1.7 mm, and 2.2 mm; (**b**) the solved result of the estimated surface shape with two images in (**a**) (the defocus amounts are −1.7 mm and 1.7 mm, respectively) is RMS = 0.047774 μm and PV = 0.25158 μm; (**c**) the solved result of the estimated surface shape with all seven images in (**a**) is RMS = 0.04484 μm and PV = 0.23882 μm; and (**d**) the difference between the measurement results with two images and with all seven images.

In the experiment, the beam size was limited to 10 mm by a stop. Firstly, we built the experiment system, fixed the thin measured mirror in the stage, observed the fringes in WYKO interferometer to maximize the contrast of the interference fringes, added the splitting prism in the optical path, and adjusted them to be coaxial with the measured mirror and pinhole. Secondly, we adjusted the position of the lens and the camera so that the light beam reflected from the measured surface through the prism and entered into the camera. We captured seven images with the camera, and the defocus amounts were 0, ±1.2, ±1.7, and ±2.2 mm. Thirdly, we disposed the collected images with the modified PR algorithm to obtain the mirror surface information. In order to make an effective comparison with the WYKO interferometer, we did not move the position of the experimental setup and measured the deformed surface again. We first measured a reference mirror with 1/20 wave flatness to remove the system errors. The measurement data with the flat mirror are treated as the system errors and subtracted from them when measuring the free-form surfaces. The seven images with different defocuses solved by modified PR are shown in Figure 3a. Figure 3b shows the estimated surface shape recovered by the modified PR with the two images in Figure 3a. The process of obtaining Figure 3b took 5.50 s, and Figure 3c shows the estimated surface shape recovered by the modified PR with all seven images. The process of obtaining Figure 3c took 75.35 s, which means that the improved PR algorithm was 15 times as fast. Figure 3d shows the difference between the estimated surface shapes recovered by the modified PR with all seven images and the two images. We could see that the difference was very small. This experiment demonstrates that the proposed modified PR algorithm is feasible in the surface metrology.

**Figure 4:**
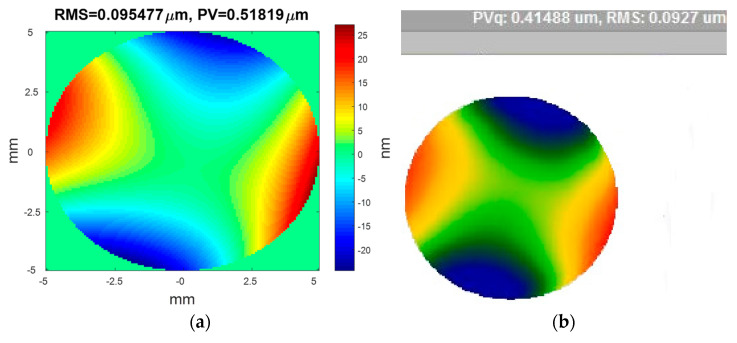
The measurement results with (**a**) PR: RMS = 0.095477 μm, PV = 0.51819 μm; (**b**) WYKO: RMS = 0.0927 μm, PV = 0.41488 μm.

To demonstrate the feasibility of the proposed method in free-form surface metrology, we apply different forces to the thin mirror, shown in Figure 2c, and take two defocused images for each force to estimate the free-form mirror surface with the improved PR algorithm. Figure 4a,b are, respectively, the mirror surface estimated by PR and the mirror surface measured with the WYKO interferometer. Comparing the results of the modified PR with the results of WYKO interferometer, the RMS difference is less than 2.777 nm, which shows that the proposed improved PR method is feasible for measuring free-form surfaces. The difference in the PV is relatively large, partially due to the following reasons. Firstly, there is a smoothing process when using the WYKO interferometer, which the solution process of the modified PR method does not have. Secondly, during the solution process we calculated the whole mask circular area with the modified PR, but Figure 4b was obtained after matting (removing boundary Burr) with the WYKO interferometer. Therefore, although the RMS of the whole mask cannot be greatly affected, it will be greatly different from the PV.

It can be seen from the above two experimental results that the process of obtaining Figure 3b from the two images took 5.50 s and the process of obtaining Figure 3c with all seven images took 75.35 s, which means that the improved PR algorithm is 15 times faster. Besides, Figure 4a,b, respectively, show the estimated surface recovered by PR and the measured surface with the WYKO interferometer; the differences between our technique and the WYKO interferometer in RMS and PV are very small, which demonstrated that our improved PR method could achieve as considerable an accuracy as the WYKO interferometer. The above two points proved the feasibility and effectiveness of our technology in the measurement of small-slope free-form surfaces.

## 4. Conclusions

In this paper, we have presented and shown experimentally with an improved PR algorithm based on the traditional gradient search algorithm to improve efficiency of phase recovery. The feasibility of the proposed method has been demonstrated by comparing the measurement results of the deformed thin mirror with the measurement results from WYKO interferometer. This work has additionally shown that PR technology is a viable and realistic method in small-slope free-form surfaces measurement. Now, we are doing research on large-slope free-form surfaces measurement with transverse translation diversity phase retrieval, and our new research will perhaps be shown in the near future.

## Data Availability

Not applicable.
